# Involvement of Phosphate and the Consequences of Its High Consumption in Energy Metabolism and Muscle Functions

**DOI:** 10.1155/jnme/5383415

**Published:** 2025-10-01

**Authors:** Nourin Jahan, Asadur Rahman, Akira Nishiyama, Kento Kitada

**Affiliations:** Department of Pharmacology, Faculty of Medicine, Kagawa University, Miki-cho, Kagawa, Japan

## Abstract

Phosphate is an important element in energy metabolism and muscle function and is essential for numerous biological processes. This review emphasizes the implications of increased phosphate intake, particularly from processed foods supplemented with phosphate additives. These dietary habits raise substantial concerns regarding their potential health effects, including the exacerbation of metabolic disorders. Elevated phosphate levels disrupt the delicate balance between energy production and utilization, markedly influencing key metabolic processes in skeletal muscle. Excessive phosphate intake may impair mitochondrial function, reduce adenosine triphosphate synthesis, and alter phosphocreatine levels, which are vital for effective muscle contraction and endurance. Additionally, chronic high-phosphate consumption has been linked to increased inflammation and oxidative stress, contributing to cardiovascular complications and muscle atrophy, especially in susceptible populations, such as those with chronic kidney disease. This review summarizes the current understanding of phosphate metabolic functions and the detrimental effects of excessive phosphate intake on energy metabolism and muscular performance. We provide insights into the adverse health effects linked to elevated phosphate levels, particularly focusing on the consequences on muscle strength and overall muscular function. In addition, we highlight the gaps in the literature and propose future studies to understand the effects of high-phosphate diets on energy metabolism, muscle function, and structural integrity through molecular processes.

## 1. Introduction

Energy metabolism is a fundamental process in living organisms and vital for activities, such as movement, temperature regulation [1], growth, and immune [2] and nervous system function [3]. It involves anabolic reactions, which synthesize new substances, and catabolic reactions, which break down substances to produce energy. Phosphate is a major component of adenosine triphosphate (ATP), an important molecule for energy storage and transfer within cells [4]. ATP hydrolyzes phosphate and converts it to adenosine diphosphate (ADP), releasing energy and fueling cellular processes, such as muscle contraction and pH regulation [5]. However, global phosphate consumption has substantially increased, doubling or tripling the recommended levels, owing to its addition to dairy and processed foods [6]. Consumption of excessive amounts of phosphate can lead to hyperphosphatemia associated with endothelial dysfunction, oxidative stress, inflammation, and cardiovascular diseases [7]. High phosphate levels impair energy metabolism and mitochondrial function, thereby reducing ATP production and muscle contraction [8]. Furthermore, excessive phosphate intake has been correlated with insulin resistance, metabolic syndrome, increased oxidative stress, and inflammation, exacerbating muscle damage and weakness [9, 10]. This review emphasizes the urgent need for a deeper understanding of how high phosphate levels modify energy metabolism, particularly to elucidate the underlying molecular mechanisms. These insights will foster future research into preventive and therapeutic interventions for metabolic disorders. Furthermore, this article explores practical dietary modifications that balance phosphate-rich foods to support muscle preservation and optimize muscle function.

## 2. The Interplay Between Energy Metabolism and Muscle Function

Skeletal muscle metabolism is highly adaptable and relies on both glucose and fatty acids, depending on the prevailing physiological conditions. At rest and during fasting, the peripheral muscle tissue primarily oxidizes fatty acids, and under such conditions, up to 80% of the energy demand may be met by fatty acid oxidation [11]. This substrate preference is modulated by intricate regulatory mechanisms, including the glucose–fatty acid cycle and insulin-mediated signaling, which ensure that energy production efficiently matches metabolic needs.

The glucose–fatty acid cycle describes the competitive relationship between fatty acids and glucose metabolism. Elevated free fatty acid (FFA) levels downregulate pyruvate dehydrogenase activity, thereby limiting the conversion of pyruvate to acetyl-CoA. This biochemical adjustment favors the oxidation of fatty acids to glucose. Insulin plays a critical modulatory role by promoting the translocation of GLUT-4 transporters to the cell membrane, thereby enhancing glucose uptake [12]. Moreover, insulin facilitates the uptake of fatty acids via proteins, such as FAT/CD36 [13], establishing a coordinated regulatory network that adjusts substrate utilization based on hormonal cues. In pathological states, such as obesity and Type 2 diabetes, insulin resistance disrupts normal substrate metabolism [14, 15]. This limitation of fatty acid oxidation promotes intracellular lipid accumulation, which exacerbates metabolic dysfunction. Empirical studies have demonstrated that a reduced capacity for fatty acid oxidation in the skeletal muscle is associated with an increased reliance on stored lipids, thereby contributing to the development of insulin resistance and hyperglycemia [16].

Exercise serves as a potent stimulus for metabolic adaptation in the skeletal muscles. During muscle contraction, catecholamine-induced lipolysis increases the availability of FFAs, while simultaneous GLUT-4 translocation augments glucose uptake [17]. The relative contributions of fat and carbohydrate oxidation varied with exercise intensity and duration. Low-intensity exercise predominantly induces fatty acid oxidation, whereas high-intensity exercise shifts the fuel preference toward glucose oxidation to meet rapid ATP demands [18].

**Figure 1:**
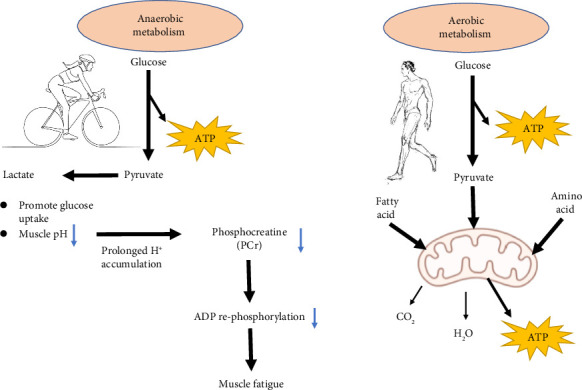
Activation of energy metabolism pathways during muscle contraction.

At the commencement of exercise, energy is immediately secured through stored ATP and phosphocreatine (PCr). However, as these energy sources are rapidly depleted, a transition toward anaerobic metabolism occurs. Glycolysis is the primary source of ATP and converts glucose to pyruvate, which is reduced to lactate under conditions of hypoxia or high-intensity exertion [19, 20]. The accumulation of lactate causes a drop in intracellular pH, typically to levels between 6.4 and 6.6, which disrupt the creatine kinase equilibrium and the capacity for rapid ATP resynthesis [21]. Acidosis exerts negative feedback on glycolytic enzymes and the PCr recovery system; the resultant increase in ADP levels is implicated in the early onset of muscle fatigue (Figure 1).

As exercise progresses from high-intensity anaerobic to prolonged aerobic conditions, there is a concomitant metabolic shift from carbohydrate to fat oxidation. In the aerobic phase, muscle glycogen and blood glucose contribute to glycolysis until their stores begin to diminish when the oxidation of fatty acids and intramuscular triacylglycerols (IMTG) becomes increasingly prominent. The citric acid cycle (TCA cycle) plays a key role during this phase. Acetyl-CoA, derived from carbohydrates, fats, and proteins, is oxidized in the TCA cycle to generate reducing equivalents (NADH and FADH_2_), which drive the electron transport chain (ETC) to synthesize ATP [22]. The efficiency of this metabolic cascade is highly dependent on cofactor availability; trace elements, such as iron, enhance the activities of citrate synthase and isocitrate dehydrogenase, thereby increasing NADH production and subsequent mitochondrial oxygen consumption [23].

During extended periods of exercise, the mobilization of FFAs from the adipose tissue significantly increases. Hormone-sensitive lipase (HSL) catalyzes the hydrolysis of IMTG, but its activity remains intact [24]. In scenarios where HSL activity is impaired, there is a compensatory reliance on carbohydrates, potentially compromising efficient transition to fatty acid oxidation. Additionally, elevated catecholamine levels during prolonged exertion promote further lipolysis and elevate plasma FFA concentrations, while simultaneously inhibiting carbohydrate oxidation via the glucose–fatty acid cycle. This shift is critical for sparing the limited glycogen stores [25].

Endurance training induces beneficial adaptations in the skeletal muscles and enhances metabolic flexibility. Regular training upregulates mitochondrial enzymes involved in β-oxidation and increases the capacity for IMTG storage and mobilization. These adaptations facilitate higher rates of lipid oxidation during submaximal exercise, thereby contributing to improved fatigue resistance and enhanced overall performance [26, 27].

Activation of energy metabolism pathways during muscle contraction involves a balance between rapid, anaerobic ATP production and slower but more efficient aerobic ATP synthesis. High-intensity contractions trigger anaerobic glycolysis, lactate accumulation, and a consequential decrease in PCr, which elevates ADP levels and contributes to muscle fatigue. In contrast, sustained exercise under aerobic conditions shifts the reliance toward mitochondrial pathways, ensuring an efficient and continuous supply of ATP essential for prolonged muscle activity.

Adequate energy availability is essential to preserve muscle strength and ensure optimal metabolic function. Insufficient energy intake reduces the synthesis of myofibrillar and sarcoplasmic proteins, ultimately leading to decreases in lean mass, disruptions in urinary nitrogen balance, reductions in free androgen index, and lower thyroid hormone concentrations [28, 29]. These impairments slow muscle repair and adaptation processes, highlighting the importance of meeting the energy requirements to sustain muscle health and functionality.

Aging and muscle strength are closely associated with energy metabolism. Muscle strength tends to decline with age, a phenomenon known as age-related muscle loss or sarcopenia [30]. Sarcopenia can begin at approximately 35 years old and typically occurs at a rate of 1%-2% a year, potentially accelerating to 3% per year after the age of 60 years, with older adults who do not do regular strength training expected to lose 1.8–2.7 kg of muscle per decade [31]. This loss of muscle mass can impact overall strength, endurance, and physical performance and may hinder everyday activities and increase the risk of falls, fractures, and mobility issues [32]. Although the loss of muscle mass is one factor contributing to this decline, energy metabolism also plays a role. Recent findings indicate that metabolism peaks earlier in life and slows down later than previously believed [33]. The specifics may vary among individuals, but metabolism generally becomes less efficient with age. This decreased efficiency can affect energy production and utilization, potentially leading to weight gain and/or difficulties in maintaining muscle mass and strength. Age-related changes in energy metabolism, such as decreased mitochondrial function and alterations in fuel utilization, can affect muscle strength [34].

## 3. Metabolic Role of Phosphate

In 1905, inorganic phosphate was shown to be necessary for alcoholic fermentation, indicating its involvement in cellular metabolism [35]. Phosphate esters were subsequently shown to enable cells to trap energy from food molecules, and high-energy phosphate groups were transferred to acceptor molecules, such as ADP, to serve as sources of chemical energy for vital cellular processes [36].

The high-energy phosphate bonds between phosphate molecules release large amounts of energy when broken. These bonds transfer chemical energy to the living organisms. The best-known high-energy phosphate bond is found in ATP, which is considered the main energy currency in cells. ATP contains two high-energy phosphate bonds, which are used to power many cellular reactions. ATP consists of three main structures: a nitrogenous base (adenine), a sugar (ribose), and a chain of three phosphate groups bound to ribose [37]. The phosphate tail of ATP is the actual power source utilized by cells. Available energy is contained in the bonds between phosphates and released when they are broken as a result of the addition of a water molecule via hydrolysis. Only the outer phosphate is usually removed from ATP to yield energy, resulting in the conversion of ATP to ADP, which contains only two phosphates. Other high-energy phosphate-containing molecules include guanosine triphosphate (GTP), cytosine triphosphate (CTP), creatine phosphate (CP), and arginine phosphate [38]. The bonds between the phosphates in these molecules are called phosphor anhydride bonds. Energy-rich phosphate esters, such as ATP, GTP, and CTP, are essential for transferring chemical energy in the body and as building blocks for DNA and RNA synthesis. Many nutrients, including glucose, fructose, galactose, glycerol, and several vitamins, are metabolized via phosphate esters [39]. Protein phosphorylation and dephosphorylation regulate the activity of many proteins, and inorganic phosphate acts as a buffer to stabilize the pH of cells and extracellular fluids [40]. Polyphosphates, which contain up to several thousand phosphates, are found in some osteoblasts and may play a role in phosphate storage, pH regulation, and protection against osmotic stress [40, 41].

## 4. Phosphate in Muscle Physiology and Functions

Phosphate plays a critical role in several physiological processes in the human body, including energy metabolism [4] and muscle function [42]. It influences various aspects of muscle physiology, including contraction, relaxation, and overall performance. Regulation of muscle function by phosphate involves several mechanisms that contribute to muscle contractility, energy production, and calcium handling.

### 4.1. Phosphate and Muscle Contraction

In muscle cells, ATP is hydrolyzed to ADP and inorganic phosphate, releasing the energy that drives muscle contraction. The release of phosphate from ATP is essential for muscle contraction, and the reuptake of phosphate is important for muscle relaxation. However, the role of phosphate in muscle contraction is complex and involves multiple pathways and regulatory mechanisms.

**Figure 2:**
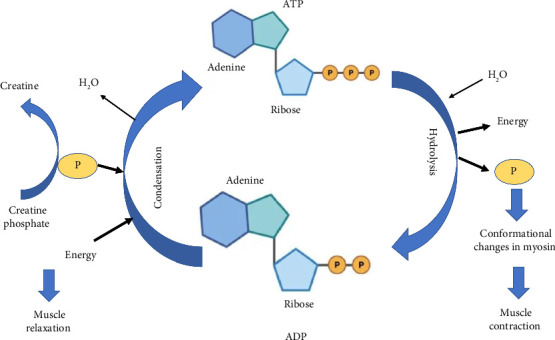
Involvement of phosphate in muscle function.

The actomyosin ATPase system is a key pathway for phosphate release during muscle contraction. This system is responsible for hydrolyzing ATP and releasing phosphate, which is then used to drive the conformational changes in the myosin head that cause muscle contraction. The rate of ATP hydrolysis is an important determinant of muscle contraction speed and force. Phosphate released from ATP also activates the sarcoplasmic reticulum (SR) calcium release channel, leading to increased intracellular calcium levels that trigger muscle contraction [43]. CP stored in the muscles acts as a reserve for high-energy phosphate groups. When ATP levels decrease during intense muscle activity, creatine kinase catalyzes the transfer of a phosphate group from ATP to creatine, forming CP. Later, during periods of high energy demand, creatine kinase can transfer the phosphate group back to ADP, regenerating ATP. This process helps sustain muscle contractions when ATP levels are temporarily low [44] (Figure 2).

ATP is hydrolyzed to ADP and inorganic phosphate, releasing the energy that drives muscle contraction. ATP hydrolysis and phosphate release contribute to the conformational changes in the myosin head, causing muscle contraction. Phosphate is taken up again during muscle relaxation. When ATP levels decrease during intense muscle activity, creatine kinase catalyzes the transfer of a phosphate group from ATP to creatine, forming CP. Creatine kinase can then transfer the phosphate group back to ADP during periods of high energy demand, regenerating ATP.

### 4.2. Phosphate and Energy Production

Phosphate contributes to ATP production via oxidative phosphorylation in mitochondria. It participates in the ETC, in which phosphate groups help transmit electrons, resulting in the formation of ATP [45]. Therefore, adequate phosphate levels are essential for optimal ATP production and ensuring sustained muscle energy.

### 4.3. Phosphate and Calcium Handling

Calcium is the key regulator of muscle contraction. When a muscle fiber generates an action potential, it triggers the release of calcium from the SR into the cytoplasm. Calcium then binds to troponin, causing a conformational change that exposes the myosin-binding sites on actin, allowing the myosin heads to form cross-bridges with actin and initiate muscle contraction. After muscle contraction, calcium was actively pumped back into the SR via ATP-dependent calcium pumps [46]. Phosphates also play a role in the regulation of calcium-dependent processes in muscle cells. Phosphate can form complexes with calcium, influencing its availability and binding to proteins involved in muscle contraction and relaxation. The interaction between phosphate and calcium helps modulate the sensitivity and responsiveness of the muscle to calcium signals [42].

### 4.4. Phosphate and Enzyme Activity

Phosphate molecules can act as cofactors or substrates for various enzymes involved in the metabolic pathways within muscle cells. The hydrolysis of ATP to ADP and inorganic phosphate is catalyzed by myosin ATPase. Phosphate groups can modify enzyme activity through phosphorylation and dephosphorylation. Phosphorylation alters the kinetic behavior of the enzyme and regulates its localization within the muscle cells, ensuring that it is available where energy is required. Phosphorylation is involved in the regulation of rabbit muscle phosphofructokinase and its interaction with F-actin, a protein involved in muscle contraction. Phosphorylated phosphofructokinase forms a more stable complex with F-actin than with its dephosphorylated form [47]. This enzymatic reaction provides the energy required for muscle contraction. Creatine kinase is responsible for the interconversion of ATP and CP. During periods of high energy demand, such as intense muscle contraction, creatine kinase catalyzes the transfer of a phosphate group from ATP to CP, converting CP to creatine and ADP to ATP. The phospholipase enzyme glucose-l-phosphoric acid (l-ester), derived from glycogen and inorganic phosphate, is found in mammalian tissues. This enzyme mediates the breakdown of glycogen, which is further metabolized to lactic acid to provide energy during strenuous activity [48]. These enzymatic modifications influence the metabolic pathways that affect muscle function, such as glycolysis and oxidative phosphorylation. Phosphate contributes to overall metabolic efficiency and energy production in muscle tissue by regulating enzyme activity.

### 4.5. Phosphate and pH Regulation

Metabolic processes, such as glycolysis and oxidative phosphorylation, generate protons as byproducts. If pyruvate production during glycolysis exceeds the oxidation level, the excess pyruvate is converted into lactic acid, which dissociates into lactate and protons. The accumulation of protons lowers the pH, potentially interfering with SR calcium release, troponin C sensitivity to calcium, and cross-bridge cycling, resulting in impaired muscle force [48]. Phosphate acts as a buffer to counteract acidosis and maintains optimal pH levels. Phosphate molecules can bind to excess protons, thereby reducing their concentration and stabilizing the pH within the muscle. This buffering capacity helps to maintain an appropriate pH range, allowing efficient muscle contraction and preventing fatigue. The ability of phosphate to accept and release protons helps regulate the acid–base balance during these metabolic reactions, preventing drastic pH changes that could compromise muscle function [49].

## 5. Effects of High Phosphate on Energy Metabolism and Muscle Functions

Hyperphosphatemia arising from underlying kidney dysfunction or excess dietary phosphate intake is significantly associated with muscle atrophy and diminished physical performance. The pathophysiology of this relationship is complex and involves several inter-related mechanisms.

Hyperphosphatemia poses substantial health risks in patients with chronic kidney disease (CKD). The compromised ability of the kidneys to excrete phosphate results in persistent hyperphosphatemia, which adversely affects muscle integrity and overall physical performance. As renal function declines and the glomerular filtration rate decreases, the capacity of the kidneys to eliminate excess phosphate diminishes, leading to the accumulation of phosphate in the bloodstream [50]. This unwanted phosphate build-up is linked to worsening CKD and correlated with detrimental cardiovascular outcomes, including vascular calcification and associated morbidity and mortality [50, 51], indicating a complex interplay between phosphate metabolism and muscle function.

Elevated phosphate levels are implicated in vascular calcification, a pathological process that considerably impairs peripheral arterial circulation, and are essential for adequate muscle function [51]. Men and women with CKD often experience muscle atrophy and decreased physical performance, which are exacerbated by the relationship between phosphate levels and skeletal muscle metabolism [50, 52]. The adverse effect on muscles is attributed to the direct role of hyperphosphatemia in causing cellular disturbances that undermine energy metabolism, which is critical for muscle contraction. These disruptions include impairments in mitochondrial function, which plays a central role in ATP production, a vital energy source for muscle activity [50, 52].

Elevated phosphate levels can alter the cellular signaling pathways necessary for energy production, resulting in diminished muscle energy balance and functionality. Furthermore, studies using animal models have indicated that prolonged high-phosphate diets lead to significant reductions in skeletal muscle mass and function, highlighting the necessity for a more extensive examination of the chronic effects of phosphate intake in human populations [53]. Even in healthy populations, high dietary phosphorus intake correlates with increased serum phosphorus levels and impaired endothelial function [54, 55]. In healthy participants, it was found that elevated serum phosphate levels correlated with unfavorable changes in vascular health, which could impair nutrient delivery to active muscles during exercise [54]. Another study demonstrated that high phosphorus intake acutely impairs endothelial function, as assessed by flow-mediated dilation (FMD). The postprandial increase in serum phosphorus was significantly correlated with the degree of FMD impairment [55]. These findings collectively suggest that even transient, postprandial hyperphosphatemia can mediate endothelial dysfunction. This may contribute to the observed link between elevated serum phosphorus and increased cardiovascular risk in healthy individuals [55]. Furthermore, this vascular dysfunction could decrease exercise capacity and promote conditions like sarcopenia, particularly in aging populations, highlighting the importance of managing dietary phosphate for maintaining muscle health. While current knowledge emphasizes the negative effects of phosphate on health, including its effects on skeletal health in patients with CKD, there is a notable gap in detailed understanding of the effects of high phosphate intake on healthy individuals and athletic populations who may have diets high in protein and, consequently, phosphates [50, 51].

For the general population, the recommended dietary allowance of phosphorus is approximately 700 mg/day. This intake supports essential physiological functions, including bone maintenance, energy metabolism, and cellular signaling [56]. However, many individuals exceed this recommendation, often due to the consumption of processed foods rich in phosphate additives. The average dietary intake ranges from 1200 to 1500 mg per day, raising concerns about potential health impacts, including cardiovascular disease and diminished bone health [57, 58]. While essential, even slight elevations in serum phosphate from high dietary intake can have significant immediate and long-term effects on energy metabolism and muscle function in healthy individuals.

In the short term, slight increases in serum phosphate may disrupt muscle energetics. Acute elevations in serum phosphorus can hinder the efficiency of PCr resynthesis, impairing energy availability during high-intensity exercise and leading to increased fatigue [59].

In the long term, chronically elevated serum phosphate is linked to adverse outcomes such as cardiovascular disease and impaired bone health, which can indirectly reduce muscle strength and increase fracture risk [57]. One potential mechanism is through impaired vascular function. As mentioned, high dietary phosphorus intake correlates with impaired endothelial function, even in healthy individuals [54, 55]. This vascular dysfunction, potentially mediated by transient postprandial hyperphosphatemia, could limit nutrient delivery to muscle, thereby decreasing exercise capacity and contributing to conditions like sarcopenia, especially in aging populations [55].

While phosphate is crucial for ATP formation, the precise threshold at which diet-induced serum phosphate changes meaningfully impact these processes in healthy individuals remains unclear. Future research should aim to establish dose–response relationships and identify the underlying mechanisms through which these subtle changes influence muscle function and metabolic efficiency.

In contrast, the dietary recommendations for patients with CKD are complex. The Kidney Disease: Improving Global Outcomes guidelines suggest that dietary phosphorus restriction is crucial for managing hyperphosphatemia, particularly in the later stages of CKD and patients undergoing dialysis [60]. The aim is to maintain serum phosphorus levels within normal limits to prevent further complications, such as mineral and bone disorders. Specifically, patients with CKD are often advised to limit their phosphorus intake to approximately 800–1000 mg/day, although the specific recommendations can vary based on the stage of kidney disease and the individual's overall health status [61].

Recent investigations have also noted the need for personalized dietary recommendations that focus on reducing phosphorus intake in healthy individuals before considerable kidney impairment occurs. Under conditions of high phosphate intake, a transient increase in serum phosphate levels can promote the formation of calcium–phosphate complexes. These complexes tend to precipitate and deposit in various soft tissues, including the vascular wall. Such deposition is a precursor to vascular calcification, as calcium phosphate accumulation triggers the osteogenic transdifferentiation of vascular smooth muscle cells, which contributes to arterial stiffening and impaired vessel function [62, 63]. These vascular changes not only alter hemodynamics, but also compromise blood flow and oxygen delivery to the skeletal muscle, thereby affecting muscle function and contractility.

The negative vascular effects of excess phosphate are accompanied by direct effects on muscle structure and performance. Elevated phosphate levels have been epidemiologically associated with decreased muscle strength and conditions, such as age-related loss of muscle strength and sarcopenia, indicating a systemic impact on musculoskeletal health [64]. At the cellular level, when muscles become fatigued, the accumulation of inorganic phosphate in the cytosol increases. This excess phosphate can infiltrate the SR and combine with calcium ions to form insoluble calcium phosphate precipitates, which inhibit the release of calcium from the SR, a critical step in excitation–contraction coupling, and thus diminish the contractile force [65]. The resulting decreases in muscle performance and strength underscore the delicate interplay between phosphate levels and muscle function.

Mitochondria, acting as cellular powerhouses, play a pivotal role in generating ATP through oxidative phosphorylation and rely on the sequential transfer of electrons along the ETC. High phosphate levels may interfere with this process by disrupting electron flow and impeding ATP production [8]. Inhibition of mitochondrial phosphate carriers prevents high phosphate–induced superoxide generation in the mitochondria [66]. Another study showed that excessive phosphate causes mitochondrial injury [67]; however, in our previous study, we did not observe any mitochondrial damage induced by high phosphate levels [68]. Moreover, elevated phosphate concentrations are associated with the suppression of myogenic differentiation, which is essential for muscle repair and regeneration. In vitro studies have indicated that elevated phosphate levels inhibit the differentiation of muscle precursor cells into mature muscle fibers, thereby impairing the ability of the body to repair damaged muscle tissue [69]. Elevated phosphate levels are associated with oxidative stress, which adversely affects the muscle cells [70]. Oxidative stress arises from an imbalance between ROS production and cellular antioxidant capacity. Under high phosphate conditions, increased ROS levels can lead to oxidative damage to proteins, resulting in their degradation through the activation of proteolytic systems, such as the ubiquitin–proteasome pathway [71]. Specifically, the upregulation of proteins, such as MuRF1 and Atrogin-1, in response to these oxidative stresses facilitates muscle atrophy [72]. This suggests that oxidative stress is a pivotal factor in the translation of high phosphate levels into muscle-wasting signals. Although the role of oxidative stress has been previously outlined, the exact mechanisms by which high phosphate levels induce these processes remain unclear. Excessive phosphate can disturb ATP production dynamics within muscle cells, potentially leading to reduced energy availability for the maintenance and repair processes that are critical for muscle health [54]. Impaired ATP synthesis has been hypothesized to contribute to muscle weakness and dysfunction; however, detailed mechanistic insights are lacking [73]. This indicates the need for further research into how phosphate dysregulation affects mitochondrial function and energy metabolism, which are essential for muscle integrity. Moreover, the activation of proteolytic enzymes in response to high phosphate may also be influenced by other pathways, such as those involving TGF-β, which has been documented to initiate oxidative stress in muscle fibers, promoting further degradation [74]. This demonstrates the interconnected nature of these cellular responses, in which phosphate levels, oxidative stress, and various signaling pathways collectively contribute to muscle atrophy.

As highlighted in the nutritional management paradigms for populations, such as those with CKD, dietary phosphate restriction is emphasized. However, comprehensive studies exploring how different dietary patterns and phosphate binders affect overall muscle health and energy metabolism are limited [75, 76]. Future research should focus on practical dietary modifications, including the balance of phosphate-rich foods, and their relationship to muscle preservation and function.

## 6. Conclusions

This review highlights the notable implications of phosphate involvement and the consequences of high phosphate consumption on energy metabolism and muscle function. Excessive phosphate intake disrupts vital metabolic pathways, leading to hyperphosphatemia and compromising the energy supply necessary for muscle contraction and cellular processes. This disruption extends mitochondrial function and integrity, affecting the ETC and ATP synthesis, and impairing cellular energy production. Furthermore, elevated phosphate levels suppress myogenic differentiation, hinder muscle repair mechanisms, and promote muscle atrophy through oxidative stress-mediated protein degradation. These findings underscore the intricate relationship between phosphate metabolism, energy homeostasis, and muscle health. Addressing phosphate imbalance through dietary interventions and therapeutic strategies may have potential in mitigating the adverse effects of high phosphate consumption on energy metabolism and muscle function, thereby contributing to the optimization of overall health and well-being. Further research is warranted to elucidate the underlying mechanisms and develop targeted interventions to preserve phosphate homeostasis and support optimal muscle function.

## Data Availability

Data sharing does not apply to this article as no datasets were generated or analyzed during the review.
